# Effects of Transcranial Direct Current Stimulation Combined With Physical Training on the Excitability of the Motor Cortex, Physical Performance, and Motor Learning: A Systematic Review

**DOI:** 10.3389/fnins.2021.648354

**Published:** 2021-04-09

**Authors:** Baofeng Wang, Songlin Xiao, Changxiao Yu, Junhong Zhou, Weijie Fu

**Affiliations:** ^1^School of Kinesiology, Shanghai University of Sport, Shanghai, China; ^2^The Hinda and Arthur Marcus Institute for Aging Research, Hebrew SeniorLife, Boston, MA, United States; ^3^Harvard Medical School, Boston, MA, United States; ^4^Key Laboratory of Exercise and Health Sciences of Ministry of Education, Shanghai University of Sport, Shanghai, China

**Keywords:** cortical excitability, motor learning, physical performance, physical training, transcranial direct current stimulation

## Abstract

**Purpose:** This systematic review aims to examine the efficacy of transcranial direct current stimulation (tDCS) combined with physical training on the excitability of the motor cortex, physical performance, and motor learning.

**Methods:** A systematic search was performed on PubMed, Web of Science, and EBSCO databases for relevant research published from inception to August 2020. Eligible studies included those that used a randomized controlled design and reported the effects of tDCS combined with physical training to improve motor-evoked potential (MEP), dynamic posture stability index (DPSI), reaction time, and error rate on participants without nervous system diseases. The risk of bias was assessed by the Cochrane risk of bias assessment tool.

**Results:** Twenty-four of an initial yield of 768 studies met the eligibility criteria. The risk of bias was considered low. Results showed that anodal tDCS combined with physical training can significantly increase MEP amplitude, decrease DPSI, increase muscle strength, and decrease reaction time and error rate in motor learning tasks. Moreover, the gain effect is significantly greater than sham tDCS combined with physical training.

**Conclusion:** tDCS combined with physical training can effectively improve the excitability of the motor cortex, physical performance, and motor learning. The reported results encourage further research to understand further the synergistic effects of tDCS combined with physical training.

## Introduction

Transcranial direct current stimulation (tDCS) is a non-invasive brain stimulation technique that modulates the neural activities of cortical brain regions by applying a constant weak current (e.g., current intensity of one electrode is usually smaller than 2 mA) *via* the scalp electrodes (Nitsche and Paulus, 2000). More and more research using tDCS has emerged in the fields of sports and rehabilitative medicine these days (Chang et al., 2017; Xiao et al., 2020). Two types of tDCS are commonly used, that is, anodal tDCS (a-tDCS) aiming to increase the excitability of the targeting cortical regions by depolarizing the resting membrane potentials of neurons, and cathodal tDCS (c-tDCS) that often induces inhibitory effects of neural excitability in the targeted brain regions (Nitsche and Paulus, 2000, 2001; Bastani and Jaberzadeh, 2012). Recent studies in the field of sports sciences have shown that using tDCS can significantly enhance physical performance, such as the toe abduction strength (Tanaka et al., 2009) and reaction time (Tseng et al., 2020) in healthy people and the knee extensor force in patients with hemiparetic stroke. Several previous systematic reviews have also confirmed that using tDCS can induce benefits to important functionalities, such as motor control, in different populations (e.g., people with Parkinson's disease (PD) (Broeder et al., 2015) and healthy cohorts (Machado et al., 2019).

More recently, researchers have started to combine tDCS with other types of interventions, such as physical (e.g., exercise, physiotherapy, etc.) and cognitive training (Beretta et al., 2020), and explore the effects of this mixed type of intervention. Studies have shown that both tDCS and exercise (e.g., strength training) can increase the excitability of cortical (e.g., primary motor cortex, M1) as measured by the amplitude of motor-evoked potential (MEP) (Kidgell et al., 2010; Mazzoleni et al., 2019). Therefore, it is speculated that this mixed-type intervention may induce greater improvements as compared to tDCS-or exercise/training-only intervention. However, a large variance in the results of the studies exploring the effects of such mixed-type intervention was observed in previous studies. For example, Kim and Ko (2013) observed that tDCS combined with grip strength training induced a greater increase in MEP amplitude as compared to tDCS applied alone, and Jafarzadeh et al. (2019) also observed that tDCS combined with physical training induced greater improvement in dynamic stability. However, Zandvliet et al. (2018) reported that in healthy people, tDCS combined with posture training did not induce significant improvement in the center of pressure (CoP) parameters when standing quietly with eyes open or closed, including the mean and variability of the amplitude of the CoP displacement and the mean and variability of the velocity of CoP fluctuation.

Therefore, the effects of this mixed-type intervention consisting of tDCS with physical training remain unclear due to the inconsistent results of previous publications. This systematic review here thus aims to examine the efficacy of tDCS combined with physical training on the excitability of M1, physical performance and motor learning function in populations without any major neurological diseases by critically evaluating and comparing the results in the publications, which will ultimately provide important knowledge to this field and informing the design of future studies.

## Methods

### Search Strategy

Literature searches were conducted up to August 2020 in electronic databases, namely, PubMed, Web of Science, and EBSCO. The following search terms were used: “transcranial direct current stimulation,” “tDCS,” “training,” “intervention,” “exercise,” “physical therapy,” and “motor learning.” In addition, the reference lists of the included articles were investigated to detect additional relevant articles that cannot be found *via* the initial electronic search strategy.

### Inclusion and Exclusion Criteria

**Figure 1:**
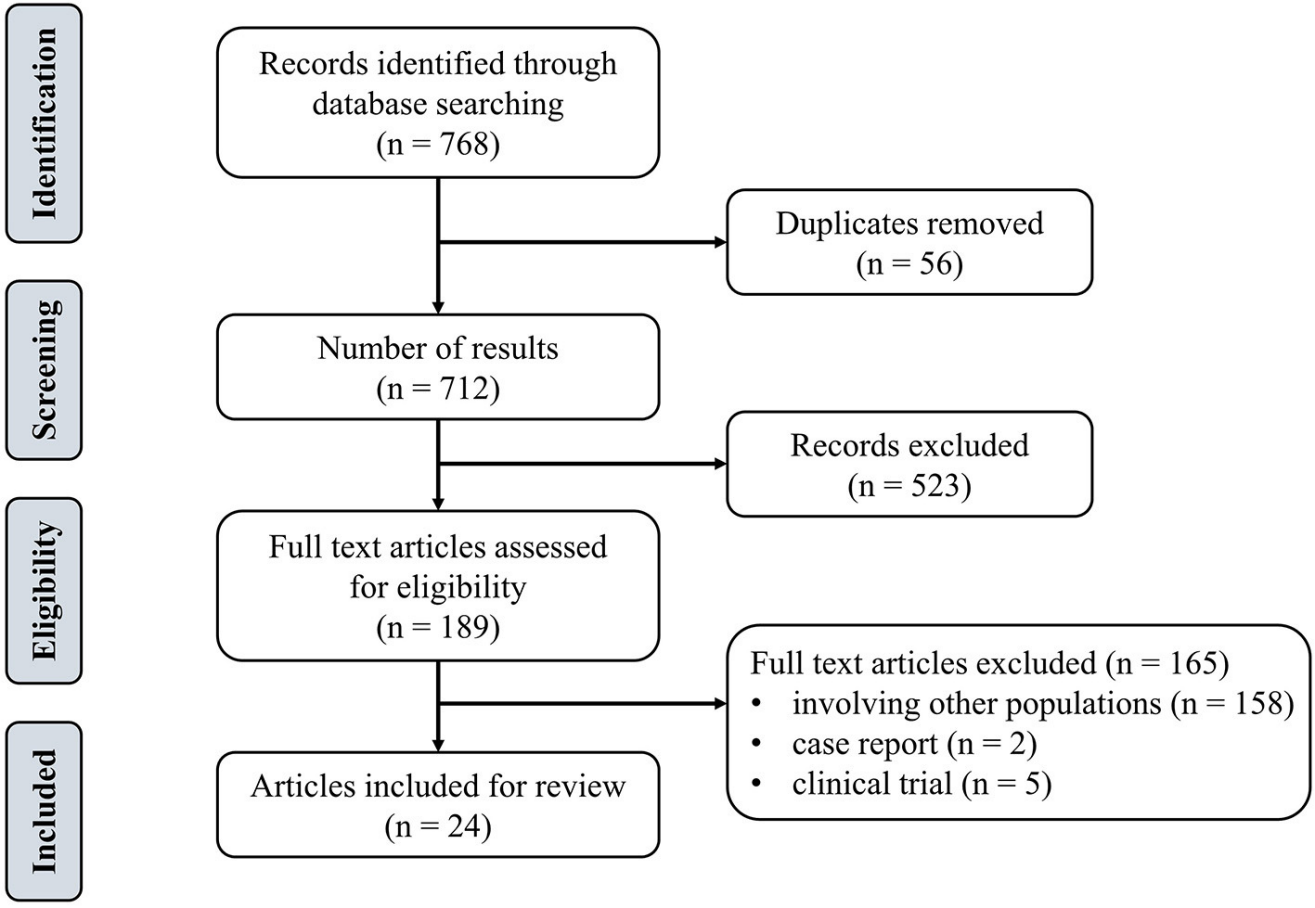
Flowchart of the search and inclusion process.

Inclusion criteria were determined on the basis of the population, intervention, comparison, and outcome approach (PICO) (Cerqueira et al., 2020). The population included adult groups (i.e., without major neurological diseases). The type of intervention was defined as tDCS combined with physical training. The control group was designed as sham tDCS (s-tDCS) combined with physical training. The outcomes included the cortical excitability as measured by MEP, dynamic posture stability index (DPSI), muscle strength, reaction time, and error rate of motor learning tasks. Exclusion criteria were: studies published in any language other than English, open-label protocol, review papers, book chapters, conference abstract, commentaries, study protocols, or clinical trial registers. Figure 1 depicted the flow diagram of the screening. Articles were then categorized in accordance with the methodological and assessed parameters.

### Data Extraction

The articles were exported to Endnote for screening and qualitative assessment. Two authors (BW and SX) independently screened all titles and abstracts. If an abstract met the inclusion criteria, the full text of the article was then reviewed for confirmation. Discrepancies were resolved by two senior investigators (WF and JZ). Then, the two authors extracted the data into spreadsheets, which included participant demographics, sample size, tDCS characteristics (e.g., electrode position, current intensity, duration), intervention characteristics (e.g., number of sessions), and main outcomes (MEP, DPSI, muscle strength, reaction time, error rate, etc.).

### Risk of Bias Assessment

We assessed the risk of bias using the Cochrane risk of bias assessment tool (Cerqueira et al., 2020). Study quality assessment included random sequence generation; allocation concealment; blinding of investigators, participants, assessors, and outcome assessors; description of losses and exclusions; selective report; and other biases. Each domain was scored as “high,” “low,” or “unclear” risk of bias.

## Results

We identified 768 studies, of which 24 meeting the eligibility criteria were included in this review. Table 1 summarized eight studies on the effects of mixed-type intervention on the excitability of M1, Table 2 summarized eight relevant studies on the effects on physical performance, and Table 3 summarized 13 studies on the effects on motor learning.

**Table 1:** The effects of tDCS combined with physical training on the excitability of the motor cortex.

**References**	**Sample size**	**Intervention**	**Control**	**Protocol**	**tDCS site**	**Combine way**	**Outcome measure**	**Time of assessment**	**Results**
Bruce et al. (2020)	26 CAI	a-tDCS + strength training	s-tDCS + strength training	10 session 18 min 1.5 mA	M1	During	MEP amplitude	Before, halfway through training (week-2), completion of training (week-4), and retention (week-6)	Cortical excitability was increased in the a-tDCS group, which lasted until the 6th week
Baltar et al. (2018)	12 YA	a-tDCS + running	c-tDCS + running s-tDCS + running	1 session 20 min 2 mA	M1	Preconditioned by tDCS	MEP amplitude	Before, immediately, 10, 20, 30, 60, and 90 min after the interventions	No significant interaction in MEP between the a-tDCS combined with running group and s-tDCS combined with running groups
Kim and Ko (2013)	44 YA	a-tDCS + grip exercise	a-tDCS s-tDCS Grip exercise	1 session 20 min 2 mA	M1	During	MEP amplitude	Before, immediately after the interventions	The combination of a-tDCS with voluntary grip exercise produced a 2-fold increase in the MEP amplitude as compared with the a-tDCS group or voluntary grip exercise group
Hendy and Kidgell (2013)	30 YA	a-tDCS + strength training	s-tDCS + strength training No intervention	9 session 20 min 2 mA	M1	During	MEP amplitude SICI	Before, immediately after the interventions	There was an increase (22.6%) in MEP amplitude for the tDCS combined with strength training group and was significantly greater than the change for the sham condition
Goodwill et al. (2013)	11 OA	Bilateral tDCS + visuomotor tracking	Unilateral tDCS + visuomotor tracking s-tDCS + visuomotor tracking	1 session 15 min 1 mA	M1	During	MEP amplitude SICI	Before, immediately and 30 min after intervention	The change from baseline to immediately after intervention for both the unilateral (38%) and bilateral (53%) conditions were significantly greater than the change for the sham condition
Karok and Witney (2013)	22 YA	Bilateral tDCS + motor sequence task	Unilateral tDCS + motor sequence task s-tDCS + motor sequence task	1 session 10 min 1.5 mA	M1	During	MEP amplitude	Before, during, immediately and 15 min after intervention	MEP amplitude of bilateral tDCS group was significantly higher than in the sham condition
Thirugnanasambandam et al. (2011)	16 YA	a-tDCS + voluntary muscle contraction	a-tDCS c-tDCS s-tDCS	1 session 20 min 1 mA	M1	Preconditioned by tDCS	MEP amplitude SICI CSP	Before, immediately after the interventions	There was no significant difference in cortical excitability between a-tDCS combined with voluntary muscle contraction group and s-tDCS group
Williams et al. (2010)	20 YA	a-tDCS + motor training	s-tDCS + motor training	1 session 40 min 1 mA	M1	During	MEP amplitude TCI	Before, immediately and 2 h after the interventions	There was a significant increase in MEP amplitude after a-tDCS combined with motor training and no significant change after sham tDCS combined with motor training

*YA, young adult; OA, old adult; CAI, chronic ankle instability; tDCS, transcranial direct current stimulation; a-tDCS, anodal tDCS; c-tDCS, cathodal tDCS; s-tDCS, sham tDCS; MEP, motor-evoked potentials; SICI, short-interval intracortical inhibition, CSP, cortical silent period; TCI, transcallosal inhibition*.

**Table 2:** The effects of tDCS combined with physical training on physical performance.

**References**	**Sample size**	**Intervention**	**Control**	**Protocol**	**tDCS site**	**Combine way**	**Outcome measure**	**Time of assessment**	**Results**
Bruce et al. (2020)	26 CAI	a-tDCS + strength training	s-tDCS + strength training	4 session 18 min 1.5 mA	M1	During	DPSI Muscle activation	Before, halfway through training (week-2), completion of training (week-4), and retention (week-6)	Dynamic balance and muscle activation improved in the a-tDCS combined strength training group from baseline to week-6
Jafarzadeh et al. (2019)	38 LBP	a-tDCS + postural training	s-tDCS + postural training Postural training	6 session 20 min 2 mA	M1	During	DPSI BBS VAS	Before, immediately and 1-month after the interventions	The postural stability indices, BBS and VAS scores significantly improved immediately and one-month after the intervention in the a-tDCS combined training group, while there were significant differences between active a-tDCS and other two groups
Zandvliet et al. (2018)	10 OA	a-tDCS + postural training	s-tDCS + postural training	1 session 20 min 1.5 mA	Cerebellum	During	CoP VAS	Before, immediately after the interventions	No significant changes in CoP comp-score and performance on the tracking task
Yosephi et al. (2018)	65 OA	M1 a-tDCS + postural training Bilateral cerebellar a-tDCS + postural training	Sham a-tDCS + Postural training Cerebellar a-tDCS postural training	6 session 20 min 2 mA	M1 Cerebellum	During	DPSI BBS	Before, immediately after the interventions	Simultaneous postural training with M1 or bilateral cerebellar a-tDCS significantly improved postural stability indices and BBS scores. Moreover, two weeks postural training alone or cerebellar a-tDCS alone is not an adequate intervention to improve the postural stability indices
Washabaugh et al. (2016)	22 YA	a-tDCS + intermittent quadriceps activity	a-tDCS + resting	1 session 20 min 1.5 mA	M1	During	Knee extension torque Knee flexion torque	Before, immediately, 5 and 25 min after the interventions	The tDCS combined with training group produced greater knee extension torques when compared with the tDCS-resting group
Steiner et al. (2016)	30 YA	a-tDCS + postural training	s-tDCS + postural training	1 session 20 min 2 mA	Cerebellum	During	Balance time Platform angle	Before, immediately, and 25 h after the interventions	Cerebellar tDCS did not improve a complex whole body dynamic balance performance in young and healthy subjects
Hendy and Kidgell (2013)	30 YA	a-tDCS + strength training	s-tDCS+ strength training No intervention	9 session 20 min 2 mA	M1	During	Maximal voluntary Strength Muscle thickness	Before, immediately, after the interventions	Maximal voluntary strength increased in both the tDCS and sham groups. There was no difference in strength gain between the two groups and no change in muscle thickness
Williams et al. (2010)	20 YA	a-tDCS + motor training	s-tDCS + motor training	1 session 40 min 1 mA	M1	During	Hand function test	Before, immediately and 2 h after the interventions	There was a larger increase in motor performance for the a-tDCS group compared with the s-tDCS group

*YA, young adult; OA, old adult; CAI, chronic ankle instability; LBP, low back pain; tDCS, transcranial direct current stimulation; a-tDCS, anodal tDCS; c-tDCS, cathodal tDCS; s-tDCS, sham tDCS; M1, Primary motor cortex, BBS, Berg Balance Scale; VAS, Visual Analog Scale; CoP, Center of pressure*.

**Table 3:** The effects of tDCS combined with physical training on motor learning.

**References**	**Sample size**	**Intervention**	**Control**	**Protocol**	**tDCS site**	**Combine way**	**Outcome measure**	**Time of assessment**	**Results**
Chen et al. (2020)	100 YA	a-tDCS + motor sequence task	s-tDCS + motor sequence task	1 session 15 min 1.5 mA	M1	Stimulation after motor training	RT	Before, during, 15 and 120 min after intervention	No significant interaction between anodal and sham conditions in motor sequencing task
Tseng et al. (2020)	20 YA	a-tDCS + stepping task	s-tDCS + stepping task	1 session 20 min 2 mA	M1	During	RT Movement time Step accuracy Step termination	Before, during, immediately and 30 min after intervention	A significant decrease in RT at 30 min after the intervention in the a-tDCS group
Rumpf et al. (2017)	47 OA	a-tDCS + motor sequence task	s-tDCS+ motor sequence task	1 session 15 min 1 mA	M1	Stimulation after motor training	Speed Error Performance index, PI	Before, immediately, 8 and 24 h after intervention	A-tDCS led to performance improvements at 8 h after the intervention and were maintained on the next day
Naros et al. (2016)	50 YA	Bilateral stimulation + motor task	a-tDCS + motor task s-tDCS + motor task	1 session 20 min 1 mA	M1	During	Motor performance	Before, immediately after the intervention	Only the bilateral paradigms led to an improvement of the final motor performance at the end of the training period as compared to the sham condition
Looi et al. (2016)	30 YA	a-tDCS + adaptive video game	s-tDCS + adaptive video game	1 session 30 min 2 mA	DLPFC	During	RT Accuracy	Before, during, immediately and two months after the intervention	Participants who received a-tDCS performed significantly better in the RT than the sham group and all effects associated with a-tDCS remained 2 months after-intervention
Fujimoto et al. (2014)	9 YA	Dual-Hemisphere tDCS + grating orientation task	Uni-Hemisphere tDCS + grating orientation task s-tDCS + grating orientation task	1 session 20 min 2 mA	S1	During	Percentage of the correct response	Before, immediately and 30 min after intervention	The percentage of correct responses on the task during dual-hemisphere tDCS was significantly higher than that in the uni-hemisphere or sham tDCS conditions
Reis et al. (2013)	109 YA	a-tDCS + visual isometric pinch force skill task	s-tDCS + visual isometric pinch force skill task	3 session 20 min 1 mA	M1	During	Motor skill measure	Before, 15 min, 3 and 6 h after intervention	Compared with the s-tDCS group, the a-tDCS group showed a significant skill improvement at 3 and 6 h after the intervention
Goodwill et al. (2013)	11 OA	Bilateral tDCS + visuomotor tracking	Unilateral tDCS + visuomotor tracking s-tDCS + visuomotor tracking	1 session 15 min 1 mA	M1	During	Tracking error	Before, immediately and 30 min after intervention	Unilateral and bilateral tDCS decreased tracking error by 12–22% at both time points and were significantly lower than the s-tDCS group
Karok and Witney (2013)	22 YA	Bilateral tDCS + motor sequence task	Unilateral tDCS + motor sequence task s-tDCS + motor sequence task	1 session 10 min 1.5 mA	M1	During	RT Mean accuracy	Before, during, immediately and 15 min after intervention	Task-concurrent stimulation with a dual M1 montage significantly reduced RTs by 23% as early as with the onset of stimulation with this effect increased to 30% at the final measurement
Stagg et al. (2011)	22 YA	a-tDCS + reaction time task	a-tDCS c-tDCS s-tDCS	1 session 10 min 1 mA	M1	During	RT	Before, immediately after the intervention	Mean RT showed a significant decrease after a-tDCS
Kang and Paik (2011)	11 YA	Bilateral tDCS + motor sequence task	Unilateral tDCS + motor sequence task s-tDCS + motor sequence task	1 session 20 min 2 mA	M1	During	RT Motor sequence task performance	Before, immediately and 24 h after intervention	Mean RT showed a significant decrease after uni-tDCS and bi-tDCS
Reis et al. (2009)	24 YA	a-tDCS + sequential visual isometric pinch task	s-tDCS + sequential visual isometric pinch task	5 session 20 min 1 mA	M1	During	Error rate Skill measure	Before, during, immediately, 8, 15, 29, 57, and 85 days after the intervention	There was greater total (online plus offline) skill acquisition with anodal tDCS compared to sham
Vines et al. (2008)	16 YA	Bilateral tDCS + motor sequence task	Unilateral tDCS + motor sequence task s-tDCS + motor sequence task	1 session 20 min 1 mA	M1	During	Percentage of change in performance Scores	Before, immediately after the intervention	Dual-hemisphere stimulation improved performance significantly more than both uni-hemisphere and sham stimulation.

*YA, young adult; OA, old adult; a-tDCS, tDCS, transcranial direct current stimulation; anodal tDCS; c-tDCS, cathodal tDCS; s-tDCS, sham tDCS; M1, Primary motor cortex; S1, somatosensory cortex; DLPFC, dorsolateral prefrontal cortex; RT, reaction time*.

### Risk of Bias Assessment

**Figure 2:**
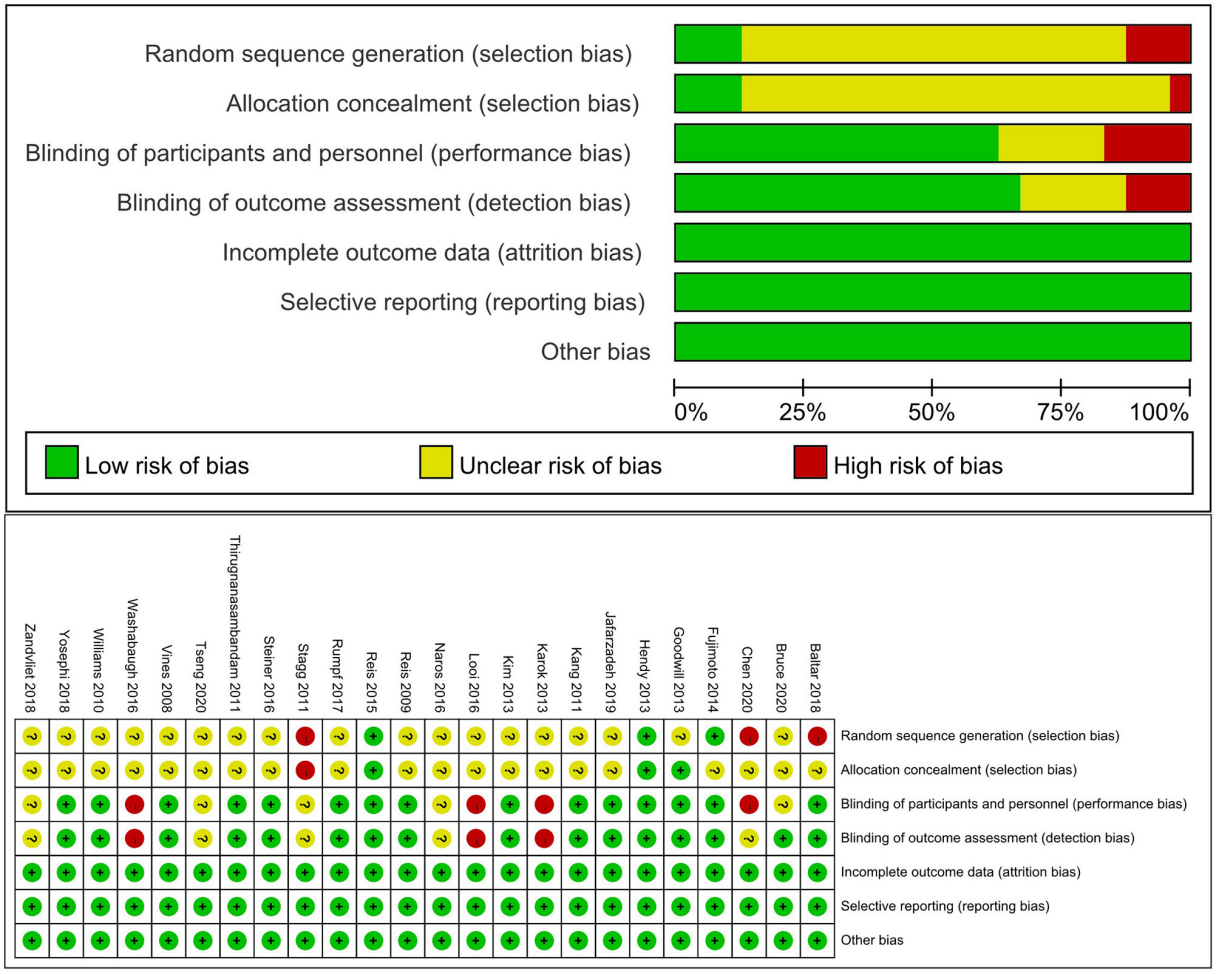
Risk of bias assessment.

The level of the risk of bias varied across studies, as shown in Figure 2. The most common potential causes of bias were inadequate description of randomization and concealment of procedures reported in studies and deemed to moderate to high risk of bias. Fifteen studies implemented a double-blinded design (Vines et al., 2008; Reis et al., 2009, 2013; Williams et al., 2010; Kang and Paik, 2011; Thirugnanasambandam et al., 2011; Goodwill et al., 2013; Hendy and Kidgell, 2013; Kim and Ko, 2013; Fujimoto et al., 2014; Steiner et al., 2016; Rumpf et al., 2017; Baltar et al., 2018; Yosephi et al., 2018; Jafarzadeh et al., 2019), in which both the study personnel and participants were not aware of the types of intervention. Five studies used a single-blinded design (Stagg et al., 2011; Naros et al., 2016; Zandvliet et al., 2018; Bruce et al., 2020; Tseng et al., 2020), and the blinding was not reported in other four studies (Karok and Witney, 2013; Looi et al., 2016; Washabaugh et al., 2016; Chen et al., 2020).

### Study Characteristics

The total number of recruited participants was 784 (462 males and 322 females, age range: 18–80 years). All interventions were designed as tDCS combined with physical training [muscle strength training (*n* = 153), balance training (*n* = 133), running (*n* = 12), shaping task (*n* = 20), visuomotor tracking (*n* = 239), grating orientation task (*n* = 9), motor sequence task (*n* = 218)]. For the design of tDCS, 19 studies used tDCS to target the M1 (Vines et al., 2008; Reis et al., 2009, 2013; Williams et al., 2010; Kang and Paik, 2011; Stagg et al., 2011; Thirugnanasambandam et al., 2011; Goodwill et al., 2013; Hendy and Kidgell, 2013; Karok and Witney, 2013; Kim and Ko, 2013; Naros et al., 2016; Washabaugh et al., 2016; Rumpf et al., 2017; Baltar et al., 2018; Jafarzadeh et al., 2019; Bruce et al., 2020; Chen et al., 2020; Tseng et al., 2020), two studies targeted the cerebellum (Steiner et al., 2016; Zandvliet et al., 2018), one study targeted both the M1 and the cerebellum (Yosephi et al., 2018), one study targeted the primary somatosensory cortex (Fujimoto et al., 2014), and one study targeted the dorsolateral prefrontal cortex (Looi et al., 2016). The current intensities of tDCS were between 1.5 and 2 mA, and the duration of each stimulation session was between 15 to 20 min. The size of the electrodes was between 35 and 40 cm^2^. Most studies used sham protocol as the control, in which the current was delivered only during the initial period of each session (i.e., the first 15 to 30 s) and then turned off (Vines et al., 2008; Reis et al., 2009, 2013; Williams et al., 2010; Kang and Paik, 2011; Stagg et al., 2011; Thirugnanasambandam et al., 2011; Goodwill et al., 2013; Hendy and Kidgell, 2013; Karok and Witney, 2013; Kim and Ko, 2013; Fujimoto et al., 2014; Looi et al., 2016; Naros et al., 2016; Steiner et al., 2016; Washabaugh et al., 2016; Rumpf et al., 2017; Baltar et al., 2018; Yosephi et al., 2018; Zandvliet et al., 2018; Jafarzadeh et al., 2019; Chen et al., 2020; Tseng et al., 2020); One study used the sham protocol that delivered current during the initial 120 s (Bruce et al., 2020).

Different timing of applying tDCS was used. Two studies applied tDCS before physical training (Thirugnanasambandam et al., 2011; Baltar et al., 2018), and two studies conducted tDCS after physical training (Rumpf et al., 2017; Chen et al., 2020); The other 20 studies applied tDCS during the physical training (Vines et al., 2008; Reis et al., 2009, 2013; Williams et al., 2010; Kang and Paik, 2011; Stagg et al., 2011; Goodwill et al., 2013; Hendy and Kidgell, 2013; Karok and Witney, 2013; Kim and Ko, 2013; Fujimoto et al., 2014; Looi et al., 2016; Naros et al., 2016; Steiner et al., 2016; Washabaugh et al., 2016; Yosephi et al., 2018; Zandvliet et al., 2018; Jafarzadeh et al., 2019; Bruce et al., 2020; Tseng et al., 2020).

Eighteen studies examined the acute effects of one session of intervention (Reis et al., 2009; Williams et al., 2010; Kang and Paik, 2011; Stagg et al., 2011; Thirugnanasambandam et al., 2011; Goodwill et al., 2013; Karok and Witney, 2013; Kim and Ko, 2013; Fujimoto et al., 2014; Looi et al., 2016; Naros et al., 2016; Steiner et al., 2016; Washabaugh et al., 2016; Rumpf et al., 2017; Baltar et al., 2018; Zandvliet et al., 2018; Chen et al., 2020; Tseng et al., 2020); six studies examined the longer-term effects of multiple-session intervention, including three (Reis et al., 2013), five (Reis et al., 2009), six (Yosephi et al., 2018; Jafarzadeh et al., 2019), nine (Hendy and Kidgell, 2013), and 10 sessions of intervention (Bruce et al., 2020). Most studies observed no side effects associated with the intervention (Vines et al., 2008; Williams et al., 2010; Kang and Paik, 2011; Stagg et al., 2011; Thirugnanasambandam et al., 2011; Goodwill et al., 2013; Hendy and Kidgell, 2013; Karok and Witney, 2013; Kim and Ko, 2013; Fujimoto et al., 2014; Looi et al., 2016; Naros et al., 2016; Steiner et al., 2016; Washabaugh et al., 2016; Rumpf et al., 2017; Baltar et al., 2018; Yosephi et al., 2018; Zandvliet et al., 2018; Jafarzadeh et al., 2019; Bruce et al., 2020; Chen et al., 2020; Tseng et al., 2020), whereas two studies reported mild discomfort, which was described as a tingling sensation (Reis et al., 2009, 2013).

### Effects of tDCS Combined With Physical Training on Cortical Excitability

Four out of eight studies observed the increase of MEP after one-session tDCS combined with physical training (including shaping tasks, visuomotor tracking, motor sequence task, and grip exercise) (Williams et al., 2010; Goodwill et al., 2013; Karok and Witney, 2013; Kim and Ko, 2013). Another two studies showed no significant improvements in MEP after a-tDCS combined with physical training (including voluntary muscle contraction and running) as compared to the control (Thirugnanasambandam et al., 2011; Baltar et al., 2018). Additionally, the other two studies observed the improved effects of multiple sessions of tDCS combined with physical training (including eccentric ankle strength training and wrist extensors strength training) on MEP (Hendy and Kidgell, 2013; Bruce et al., 2020). Moreover, Bruce et al. (2020) reported that such increase of MEP can last for 2 weeks (Table 1).

### Effects of tDCS Combined With Physical Training on Physical Performance

Three out of eight studies reported beneficial effects of tDCS combined with physical training on postural control, that is, the decrease of DPSI (Yosephi et al., 2018; Jafarzadeh et al., 2019; Bruce et al., 2020). Another two studies that assessed postural control reported no significant difference between anodal and s-tDCS at post-test (Steiner et al., 2016; Zandvliet et al., 2018). One study observed the improvement in shaping task performance at post-test (Williams et al., 2010). One study reported significantly increased knee extension moment (Washabaugh et al., 2016), and another study reported no significant difference in dynamic 1RM strength between anodal and s-tDCS at post-test (Hendy and Kidgell, 2013) (Table 2).

### Effects of tDCS Combined With Physical Training on Motor Learning

Twelve out of 13 studies observed that one session of tDCS combined with physical training improved the motor learning performance, as assessed by decreased reaction time (Stagg et al., 2011; Karok and Witney, 2013; Looi et al., 2016; Tseng et al., 2020) and error rate (Goodwill et al., 2013; Fujimoto et al., 2014), increased keystroke rate (Vines et al., 2008), and improved task performance (Reis et al., 2009, 2013; Kang and Paik, 2011; Naros et al., 2016; Rumpf et al., 2017). The other study that assessed reaction time on a motor sequence task reported no significant difference in the a-tDCS group as compared to the sham group (Chen et al., 2020).

## Discussion

To the best of our knowledge, this systematic review is the first to assess the effects of tDCS combined with physical training on the excitability of M1, physical performance, and motor learning performance. Our results showed that tDCS combined with physical training can induce significant increase in MEP (75%, 6 out of the 8 included studies), improvement in physical performance (62.5%, 5 of the 8 included studies), and motor learning (92.3%, 12 of the 13 included studies).

### Cortical Excitability

Studies have shown tDCS itself holds great promise to improve functional performance and help in rehabilitative medicine field *via* the facilitation and modulation of cortical excitability and plasticity (Santos Ferreira et al., 2019). However, large inter-personal variability in the effects of tDCS is observed due to the variance in the protocol of using tDCS, including the electrode position, dose (current intensity and duration), and differences in the brain structure across people (e.g., skull thickness, subcutaneous fat levels, cerebrospinal fluid density, cortical surface topography, age, gender, and genetics) (Wiethoff et al., 2014). Fritsch et al. (2010) reported tDCS combined additional synaptic activation (e.g., physical training) may lead to synapse specificity as a source for changes in synaptic strength, which provides an evidence for the cellular and molecular mechanisms of tDCS combined additional synaptic activation. Therefore, researchers are expected to produce more stable and effective effects to improve cortical excitability by combining tDCS (exogenous neuromodulation) with physical training (endogenous neuronal activation) (Bliss and Cooke, 2011). Six out of the eight included studies reported that a-tDCS combined with physical training induces a greater extent in the increase of cortical excitability as compared to control (i.e., sham plus physical training). This augmentation of mix-type intervention may arise from the increased synaptic strength *via* modulating the activity of N-methyl-D-aspartic acid and γ-aminobutyric acid receptors (Samii et al., 1996; Liebetanz et al., 2002; Nitsche et al., 2012). Previous studies have also shown that compared to using physical training only, tDCS combined with physical training can induce a greater increase in synaptic strength in human (Samii et al., 1996; Bliss and Cooke, 2011). Additionally, a functional magnetic resonance imaging (*f* MRI) study that conducted a-tDCS combined with hand movements found that a-tDCS application during motor tasks enhances voxel counts, peak intensity, and cortical activation on the targeted motor cortex compared with the same motor task only without using tDCS (Kwon and Jang, 2011). Taken together, a-tDCS combined with physical training may augment the increase of cortical excitability as compared to using one type of intervention only. There is evidence that the temporary modifications in cortical function correspond with transient effects in motor behaviors (Hendy and Kidgell, 2013). Meanwhile, Fregni et al. (2006) reported a significant correlation between motor function improvement after M1 a-tDCS and MEP increase.

It should be noted that 2 out of the 8 studies observed non-significant effects of intervention on cortical excitability. This may be due to two potential reasons, that is the timing of the administration of tDCS and the intensity of the physical training. These two studies administrated tDCS before physical training (Thirugnanasambandam et al., 2011; Baltar et al., 2018). Remarkably, the two studies reported similar results: physical training reduces the enhancement effect of a-tDCS on motor cortical excitability or even decreases the cortical excitability. This phenomenon may be related to the timing of tDCS administration. Homeostatic plasticity describes the fact that neuroplastic excitability diminutions are more easily achieved in highly active cortical networks but are more difficult to achieve in networks with low-level activity (Thirugnanasambandam et al., 2011). The application of a-tDCS enhances the level of motor cortical excitability, and the subsequent physical training may induce the depotentiation phenomenon. These specific mechanisms of depotentiation have been proven in animal experiments (Kumar et al., 2007). Regarding to the intensity of the physical training, Baltar et al. (2018) reported that high-intensity physical activity (e.g., running with a heart rate at 77–95% of the maximum) may cause fatigue and decrease cortical excitability, while moderate-intensity physical activity (e.g., running with a heart rate at 64–76% of the maximum) increases cortical excitability. This is also consistent with previous study showing that the task characteristics play a major role in determining the resulting plasticity (Bolognini et al., 2009). Therefore, the negative results of Baltar et al. (2018) and Thirugnanasambandam et al. (2011) may relate to the timing of tDCS administration and the characteristics of training protocols.

### Physical Performance

Five of the eight included studies reported that a-tDCS combined with physical training improved physical performance more than the s-tDCS combined with physical training. Although the other three studies did not report that a-tDCS combined with physical training was superior to s-tDCS combined with physical training, it still showed certain positive effects. For example, Hendy and Kidgell (2013) observed that a-tDCS combined with physical training induced 14.89% increase in wrist strength. Findings from Yosephi et al. (2018) and Jafarzadeh et al. (2019) also indicated a higher effect of a-tDCS combined with physical training compared with the application of physical training alone. In the cognitive domain, a combination of tDCS and aerobic training could act synergistically to improve cognitive performance beyond the level known for each technique alone (Steinberg et al., 2018). Therefore, the combination of tDCS and physical training may play a synergistic role in improving physical performance. The reason for the improvement in physical performance may be caused by cortical excitability. Although many studies have proven that tDCS combined with physical training can increase cortical excitability and improve physical performance, none has explored the correlation between cortical excitability and physical performance. Future research should conduct a correlation analysis between cortical excitability and physical performance to clarify the relationship.

Although several studies (Hendy and Kidgell, 2013; Steiner et al., 2016; Zandvliet et al., 2018) found that a-tDCS combined with physical training could significantly improve physical performance, no significant differences in strength of wrist extensors and dynamic balance task performance were observed in comparison with s-tDCS combined with physical training. Healthy participants performed well and may experience a pronounced “ceiling effect.” These individuals reached their maximum potentials after the training, leaving less room for the desired improvement in physical performance by a-tDCS combined with physical training (Chen et al., 2020). Yosephi et al. (2018) and Steiner et al. (2016) also reflected this problem. The DPSI of healthy elderly people decreased after the combination training, whereas similar results of the balance time of healthy young people were obtained for anodal and s-tDCS after the intervention (Steiner et al., 2016; Yosephi et al., 2018). This result indicates that the effect of a-tDCS combined with physical training may be disrupted by the “ceiling effect.”

### Motor Learning

The acquisition and consolidation of motor skills are crucial for sports or clinical areas; therefore, strategies to improve motor skill learning are important (Reis et al., 2013). In the past decade, tDCS has been frequently used to promote motor skill learning as a neuroregulatory approach. a-tDCS can promote motor skill acquisition (Vines et al., 2008; Reis et al., 2009, 2013; Kang and Paik, 2011; Stagg et al., 2011; Goodwill et al., 2013; Karok and Witney, 2013; Fujimoto et al., 2014; Looi et al., 2016; Needle et al., 2017) and improve translation into stable performance for days (Reis et al., 2009, 2013; Kang and Paik, 2011; Goodwill et al., 2013; Karok and Witney, 2013; Looi et al., 2016; Rumpf et al., 2017; Tseng et al., 2020).

tDCS may influence motor learning behavior by regulating excitability and synaptic plasticity in interest regions (Goodwill et al., 2013). A study on the motor learning network showed that converging activations are revealed in the dorsal premotor cortex, supplementary motor cortex, primary motor cortex, primary somatosensory cortex, superior parietal lobule, thalamus, putamen, and cerebellum during the movement learning process (Hardwick et al., 2013). A previous study on combining tDCS and *f* MRI showed that a-tDCS over M1 during motor skill learning leads to regional cerebral blood increase in M1 and other brain regions (e.g., the caudal portion of the anterior cingulate cortex, right parieto-occipital junction) (Lang et al., 2005). Furthermore, previous studies have reported that the functional connectivity within the motor network increases (Amadi et al., 2014), and the interhemispheric inhibition is reduced (Goodwill et al., 2013) after a-tDCS over M1. Therefore, the combination of a-tDCS with physical training promotes the acquisition and consolidation of motor skills by modulating the excitability of M1, as well as the regions that are related to the motor learning.

### Limitations

Firstly, it is still challenging to determine the optimal montage of tDCS (e.g., duration, number of sessions, appropriate cortical targets) and the optimal design of the mixed-type intervention (e.g., the most appropriate type of the physical training program) for increasing cortical excitability, improving physical performance, and augmenting motor learning due to the large variance of study protocols in current publications. Second, this review focuses on the effects of the intervention on healthy cohorts, without limiting other demographic factors (i.e., young vs. old adults), which may potentially contribute to the variance of tDCS-induced effects. Lastly, some factors limited our ability to draw more accurate conclusions about the synergistic effects of tDCS combined with physical training; for example, only a few studies assessed the effects on single-joint or multi-joint strength and endurance.

## Conclusion

This systematic review shows that compared with s-tDCS combined with physical training, a-tDCS combined with physical training has a greater effect on the excitability of the motor cortex, physical performance, and motor learning, including increased MEP, improved dynamic balance performance, and decreased reaction time. tDCS combined with physical training may promote benefits to a great extent on synaptic intensity and brain functional connectivity beyond the level known for each technique alone. However, further studies are needed to explore the most potentially effective physical training protocol and the differences among populations at different physiological levels.

## Data Availability Statement

The original contributions presented in the study are included in the article/supplementary material, further inquiries can be directed to the corresponding author/s.

## Author Contributions

BW, WF, and JZ contributed to conception and design of the study. BW organized the database and wrote the first draft of the manuscript. All authors contributed to manuscript revision, read, and approved the submitted version.

## Conflict of Interest

The authors declare that the research was conducted in the absence of any commercial or financial relationships that could be construed as a potential conflict of interest.
